# Altered cerebrospinal fluid levels of kallikrein 5 (KLK5) in Alzheimer's disease

**DOI:** 10.1002/alz70856_103738

**Published:** 2025-12-26

**Authors:** Barbara Mroczko, Agnieszka Kulczynska‐Przybik, Daria Krawczuk, Renata Borawska, Izabela Winkel

**Affiliations:** ^1^ Department of Neurodegeneration Diagnostics, Medical University of Bialystok, Bialystok, Poland; ^2^ Dementia Disorders Centre, Medical University of Wroclaw, Scinawa, Poland

## Abstract

**Background:**

Alzheimer's disease (AD) constitutes an important global health challenge. Understanding the various molecular mechanisms underlying the pathogenesis of the disease is crucial for the efficient treatment of the disease. Kallikrein‐related peptidases (KLKs), a family of serine proteases, have gained interest as potential biomarkers and therapeutic targets for AD. It is reported that the kalikrein family proteins may have amyloidogenic potential in the brain and KLKs may contribute to the pathogenesis of AD. In the available literature, some KLKs such as KLK6 and KLK8 were more extensively tested and are discussed as potential therapeutical targets, whereas there is a lack of information about KLK5 in AD. Given that, we investigated the KLK5 in cerebrospinal fluid (CSF) of patients with AD and explored associations with established AD biomarkers.

**Method:**

The concentrations of KLK5 as well as classical CSF biomarkers, such as amyloid beta 1‐42 (Aß‐42), amyloid beta 1‐40, Tau, as well as pTau181, were determined in cerebrospinal fluid patients with AD and subjects without cognitive decline by multiplexing, and enzyme‐linked immunosorbent methods.

**Result:**

Significantly higher CSF concentrations of KLK5 were found in AD patients compared to the subjects without cognitive decline. Furthermore, in the group of patients with AD, the levels of KLK5 significantly correlated with the CSF amyloid beta ratio.

**Conclusion:**

Our findings indicate that kallikrein 5 may play a role in the pathogenesis of AD, particularly in the amyloid pathology. KLKs may serve as additional indicator in combination with classical well‐known biomarkers such as Aβ and tau proteins.

